# The global prevalence of methicillin-resistant *Staphylococcus aureus* colonization in residents of elderly care centers: a systematic review and meta-analysis

**DOI:** 10.1186/s13756-023-01210-6

**Published:** 2023-01-29

**Authors:** Amir Hossein Hasanpour, Mahdi Sepidarkish, Abolfazl Mollalo, Ali Ardekani, Mustafa Almukhtar, Amal Mechaal, Seyed Reza Hosseini, Masoumeh Bayani, Mostafa Javanian, Ali Rostami

**Affiliations:** 1 Student Research Committee, Babol University of Medical Sciences, Babol, Iran; 2 Department of Biostatistics and Epidemiology, School of Public Health, Babol University of Medical Sciences, Babol, Iran; 3 Department of Public Health and Prevention Science, School of Health Sciences, Baldwin Wallace University, Berea, OH USA; 4 School of Medicine, Shiraz University of Medical Sciences, Shiraz, Iran; 5 Harlem Medical Center, Bridgeview, IL USA; 6 Department of Hematopoietic Biology and Malignancy, The University of Texas Md Anderson Cancer Center, Houston, TX USA; 7 Social Determinants of Health Research Center, Health Research Institute, Babol University of Medical Sciences, Babol, Iran; 8 Infectious Diseases and Tropical Medicine Research Center, Health Research Institute, Babol University of Medical Sciences, Babol, Iran

**Keywords:** Methicillin-resistant *Staphylococcus aureus*, Residential facilities, Nursing homes, Long-term care, Systematic review

## Abstract

**Background:**

Methicillin-resistant *Staphylococcus aureus* (MRSA) is a difficult to treat infection, particularly in residents of elderly care centers (ECCs). Despite the substantial burden of MRSA, an inadequate number of studies have analyzed MRSA prevalence in ECCs.

**Objectives:**

We conducted a worldwide systematic review and meta-analysis on the prevalence and risk factors of MRSA in ECCs.

**Methods:**

We searched MEDLINE/PubMed, EMBASE, Web of Science, and Scopus databases and the gray literature sources for all studies published between January 1980 and December 2022 on the prevalence of MRSA in ECCs. A random-effects model was utilized to estimate pooled prevalence rates at 95% confidence intervals (CI). Moreover, the data were analyzed based on World Health Organization-defined regions, income, and human development index levels.

**Results:**

In total, 119 studies, including 164,717 participants from 29 countries, were found eligible for meta-analysis. The pooled global prevalence of MRSA was 14.69% (95% CI 12.39–17.15%; 16,793/164,717). Male gender [prevalence ratio (PR) = 1.55; 95% CI 1.47–1.64], previous MRSA infection (PR = 3.71; 95% CI 3.44–4.01), prior use of antibiotics (PR = 1.97; 95% CI 1.83–2.12), hospitalized within the previous year (PR = 1.32; 95% CI 1.20–1.45), have had any wound (PR = 2.38; 95% CI 2.23–2.55), have used urinary catheter (PR = 2.24; 95% CI 2.06–2.43), have used any medical device (PR = 1.78; 95% CI 1.66–1.91), and those with diabetes (PR = 1.55; CI 1.43–1.67) were more likely to be colonized by MRSA than other patients.

**Conclusion:**

Screening programs and preventive measures should target MRSA in ECCs due to the high global prevalence rates.

**Supplementary Information:**

The online version contains supplementary material available at 10.1186/s13756-023-01210-6.

## Background

The improvement of lifestyle and medical care, and declining birth rates in recent decades, have led to a rapid increase in life expectancy and the mean age of the population, especially in developed countries [1]. According to the World Health Organization (WHO) report, by 2050, it is estimated that almost more than a quarter of the world's population will be over 60 years old [2]. The number of elderly is expected to reach 1.4 billion by 2030 and 2.1 billion by 2050 [3, 4]. It is evident that a considerable portion of seniors will need intensive care, while the majority of them merely need daycare facilities [5, 6]. Therefore, the exponential need for institutions providing long-term care facilities, nursing homes, and residential care homes (all defined as elderly care centers (ECCs) in this study) is anticipated [7].

Multidrug-resistant organisms (MDROs) are among the leading causes of morbidity and mortality in ECCs [8, 9]. Elderly at ECCs are prone to colonization/infection with MDROs mainly due to age-associated morbidities (i.e., cognitive disorders), perpetual living in a confined and crowded area, prolonged and recurrent use of broad-spectrum antibiotics, and frequent referral to and from acute-care hospitals [10–12]. One of the most prevalent MDROs in ECCs is methicillin-resistant *Staphylococcus aureus* (MRSA) [8, 13, 14]. MRSA is a global health-threatening organism in healthcare settings, as it is resistant to antibiotics making the treatment more complex [15]. MRSA infection could be responsible for fatal sepsis, pneumonia, and higher rates of myocardial infarction and heart failure in patients with bacteremia [16]. A national cohort study conducted in the United States indicated that MRSA colonization among community adults aged 40–85 is associated with a significantly increased mortality risk (hazard ratio, 1.75; 95% CI 1.12–2.73) [17]. Additionally, the attributable cost of MRSA among hospitalized individuals (≥ 65 years) is estimated to be 22,293 $ per patient (95% CI 19,101–24,485$) in the United States [18]. Another cohort study in Chinese nursing homes showed that MRSA colonization was an independent risk factor for 2 year infection-related mortality (hazard ratio, 1.96; 95% CI 1.01–3.78) [19].

Considering the significant toll of MRSA, monitoring the extent of colonization, and identifying the key risk factors of MRSA acquisition in ECCs is essential for controlling and reducing the burden of this disease in ECCs residents. In the past years, a growing body of epidemiological literature evaluated the prevalence rate of MRSA colonization in ECCs from various countries; nevertheless, there is no comprehensive study to estimate global and regional prevalence rates. To bridge this gap, we performed a systematic review and meta-analysis to evaluate the worldwide prevalence and identify potential determinants of MRSA colonization in the elderly living in ECCs.

## Methods

We followed the Preferred Reporting Items for Systematic Reviews and Meta-analyses (PRISMA) guidelines to perform and report this systematic review and meta-analysis [20] and registered it in PROSPERO (CRD42021291492).

### Data source and searching strategies

Two authors (A.H.H and A.A.) independently searched MEDLINE/PubMed, EMBASE, Web of Science collection, and Scopus databases on December 20, 2021, for articles published since January 1, 1980. The search was updated for twice; first, on February 15, 2022, and second, on December 15, 2022. Moreover, grey literature was searched through manual inspections of bibliographies of retrieved studies and internet searches of Google and Google Scholar. We applied the following search terms: [(“*Staphylococcus aureus*” OR "*methicillin resistant Staphylococcus aureus*" OR "*MRSA*" OR "*multidrug-resistant organisms*" OR "methicillin resistance" OR “antibiotic-resistant infections” OR “antibiotic-resistant bacteria”) AND ("old age homes" OR "Nursing Homes" OR "homes for the aged" OR "residential facilities" OR “Residential Care Homes” OR "housing for the elderly" OR "Long term care facility") AND (“prevalence” OR “epidemiology” OR “incidence”)] (Additional file 1: Fig. S1). The search syntax was modified according to the properties of each database. We included studies in all languages, and those with languages unknown to the study team were translated into English using the “Google Translate” online tool. All articles were imported into EndNote reference manager software X8 (Thompson and Reuters, Philadelphia, USA), and duplicates were removed.

### Inclusion and exclusion criteria

Two authors (A.H.H. and M.A.) independently screened titles and abstracts of retrieved citations to identify eligible studies that met the following inclusion criteria: (i) presented data on prevalence or incidence of MRSA in ECCs; (ii) reported data on the elderly (minimum age wasn’t specified, we accepted definition of the included studies for elderlies in ECCs); (iii) included at least 30 tested elderlies. Only the last report was included in multiple sequential articles that were generated from the same data set (e.g., cohort studies). In clinical trials, we only extracted baseline data. Articles were excluded if they were (i) performed only on MRSA patients; (ii) performed on elderly in community or hospitals; (iii) included only a specific group of elderly with a specific disease or situation; (iv) used datasets that overlapped with other articles; (v) studies following the MRSA outbreaks in ECCs; (vi) case series, case reports, and articles without original data such as reviews or systematic reviews, comments, editorials, and corresponding letters.

### Data extraction and quality assessment

After screening the articles, the required data from each eligible study were extracted and imported into a standardized Microsoft Excel spreadsheet (version 2016; Microsoft Corporation, Redmond, USA). The following data were extracted from each study: name of the first author, year of publication, start and end year of study, country, type of ECCs, study design, body sampling sites, diagnostic methods for MRSA detection, age (mean and range) of tested elderlies, the total number of tested participants, the total number of participants tested positive for *Staphylococcus aureus*, methicillin-sensitive *Staphylococcus aureus* (MSSA), and MRSA. We stratified countries according to WHO-defined regions [17], World Bank's income category [21], gross national income per capita [22], and the human development index (HDI) [23]. Furthermore, to evaluate the main risk factors of MRSA prevalence, we extracted data (if available in individual studies) of gender, prior antibiotics use, prior MRSA infection, prior hospitalization, having any wound, urinary catheter, use of any medical device, diabetes, antacid use, and dementia. To conduct the quality assessment and the risk of bias in included studies, we used the Joanna Briggs Institute (JBI) Critical Appraisal Checklist for studies that reported prevalence data [24]. The detailed items of JBI tools are presented in Additional file 1: Table S1.

### Data synthesis and statistical analysis

Stata software version 16.0 (STATA Corp., College Station, Texas, USA) was used to perform the meta-analyses. Before pooling prevalence estimates, the variance of the raw prevalence from each included study was stabilized by using the Freeman-Tukey double arc-sine transformation [25]. The Cochran’s Q test and *I*^2^ index were used to calculate the between-studies heterogeneity [23, 26]. A* P*-value < 0.01 for the Cochran’s Q test and an *I*^2^ of > 75% are considered as significant and high heterogeneity, respectively [23, 26, 27]. DerSimonian and Laird random-effects model (REM) was used in case of high heterogeneity, to conservatively estimate the pooled prevalence of MRSA at 95% confidence intervals (CIs) [25, 28]. We estimated the prevalence in individual countries by synthesizing the prevalence rates of all studies from the same country. Further, we calculated the prevalence rates of MRSA for the WHO-defined regions by synthesizing the data for countries within the same region [29]. We did not assess publication bias, as in prevalence studies, there were no group comparisons or hypothesis tests of “treatment effect” [30].

We performed subgroup and meta-regression analysis to identify sources of heterogeneity, determine the risk factors and the effects of socio-demographic factors on prevalence rates. Subgroup analyses were undertaken according to WHO-defined regions, income and HDI levels, type of diagnostic methods, risk of bias, and key determinants. Corresponding prevalence ratios (PRs) were estimated for variables subjected to subgroup analysis. Meta-regressions were performed on publication year, sample size, income and HDI levels.

## Results

### Characteristics of the included studies

**Fig. 1:**
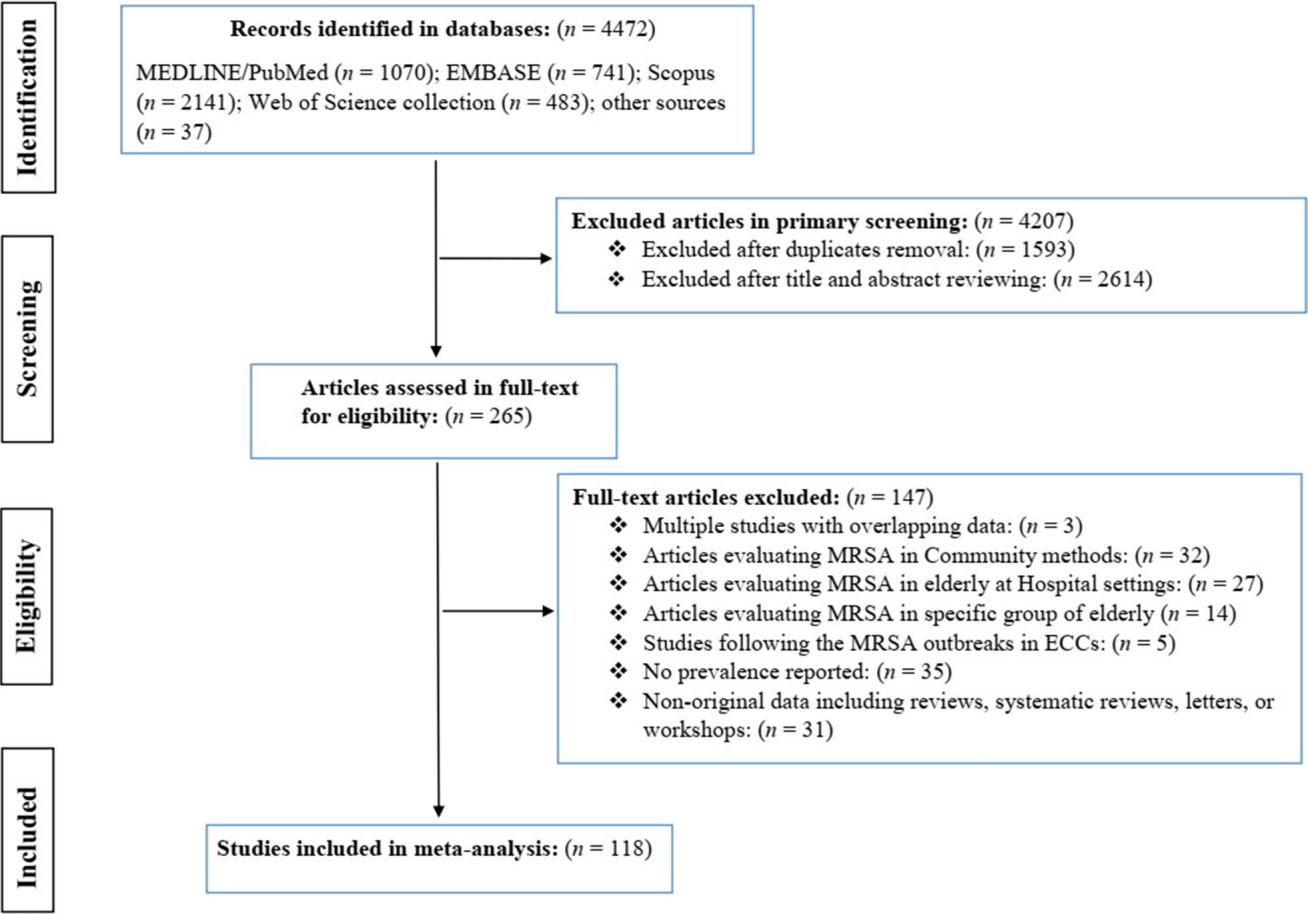
The PRISMA diagram of the study selection

As outlined in Fig. 1, a total of 4472 articles were identified in our initial literature search, of these 118 articles (119 studies) involving 164,717 participants from 29 countries were included in the meta-analysis. The main characteristics of the included studies are shown in Additional file 1: Table S1. Studies included were published between 1990 and 2022. Twenty-six studies had data to determine the proportion of MRSA and MSSA. Overall, eligible studies were available for five WHO-defined regions; 65 studies were from European region, 34 from the region of the Americas, 18 studies from the Western Pacific region, and one study for each of the Eastern Mediterranean and African regions. Countries with the most eligible studies were the United States (31 studies), China (12 studies), Germany (10 studies), the United Kingdom (eight studies), and Italy (six studies). Regarding study designs, 88 studies were cross-sectional, 21 studies were prospective cohort, seven studies were randomized controlled trial (RCT), and three studies were case control. Regarding the location of studies, 71, 41, and 7 studies were performed in nursing homes, long-term care facilities, and residential care homes, respectively. Considering the risk of bias, 68 and 51 studies were determined as the low and moderate risk of biases. All studies used culture-based methods to determine the MRSA prevalence, and 54 performed further analyses using molecular methods. Additional information is presented in Tables 1 and 2.

**Table 1:** Global and regional pooled prevalence rates of MRSA among elderly living in ECCs; results from 119 studies performed in 30 countries

WHO regions*/country	Number of datasets	Number of individuals screened (total)	Number of individuals with MRSA	Pooled prevalence % (95% CI)
Global	119	164,717	16,793	14.69 (12.39–17.15)
Americas	34	86,065	7508	22.27 (15.56–29.79)
United States	31	21,457	5517	23.78 (19.12–28.77)
Brazil	2	526	71	13.11 (10.34–16.14)
Canada	1	64,082	1920	3.01 (2.87–3.13)
Western Pacific Region	18	15,419	2936	16.57 (11.70–22.10)
China	12	11,337	2101	18.07 (11.18–26.17)
Japan	3	354	32	8.81 (5.99–12.08)
Singapore	2	3613	785	21.72 (20.39–23.08)
Australia	1	115	18	15.65 (9.55–23.60)
European region	65	62,893	6319	10.93 (8.56–13.55)
Germany	10	10,857	432	4.67 (2.59–7.29)
United Kingdom	8	8633	2037	18.66 (12.07–26.28)
Belgium	7	13,508	1403	8.97 (4.94–14.03)
Italy	6	1710	246	16.34 (10.23–23.52)
Spain	5	2700	460	15.45 (9.50–22.52)
Sweden	4	1122	139	4.52 (0.01–33.07)
Netherlands	3	5841	261	6.63 (0.01–35.64)
France	3	1500	121	13.89 (3.70–29.01)
Israel	3	545	68	14.82 (5.28–27.95)
Switzerland	3	12,128	805	13.15 (7.08–20.70)
Finland	1	213	2	0.94 (0.11–3.35)
Ireland	2	818	82	9.46 (7.51–11.61)
Poland	2	248	61	22.18 (17.16–27.62)
Slovenia	2	209	22	10.50 (6.62–15.09)
Greece	1	227	33	14.54 (10.22–19.81)
Croatia	1	877	62	7.07 (5.46–8.97)
Georgia	1	56	8	14.29 (6.38–26.22)
Austria	1	500	0	0.01 (0.00–0.74)
Luxembourg	1	954	69	7.23 (5.67–9.06)
Turkey	1	247	8	3.24 (1.41–6.28)
African region	1	152	13	8.55 (4.63–14.18)
South Africa	1	152	13	8.55 (4.63–14.18)
Eastern Mediterranean	1	188	17	9.04 (5.36–14.08)
Saudi Arabia	1	188	17	9.04 (5.36–14.08)

*The WHO regions are sorted based on prevalence rates, and countries are sorted based on the number of datasets

**Table 2:** Prevalence estimates for the MRSA in the elderly, according to the study characteristics and socio-demographic factors

Variable subgroup	Number of datasets	Number of elderlies screened (total)	Number of elderlies with MRSA	Pooled prevalence % (95% CI)
*Income*				
Upper middle	16	12,071	2193	16.46 (10.79–23.05)
High	103	152,646	14,600	14.42 (11.98–17.04)
*HDI*				
High	15	12,015	2185	16.60 (10.74–23.42)
Very high	104	152,702	14,608	14.42 (11.99–17.02)
*Type of setting*				
Long-term care facilities	41	88,088	5069	16.29 (12.29–20.71)
Nursing homes	71	69,652	10,730	14.35 (11.50–17.44)
Residential care homes	7	6977	994	9.58 (3.62–17.93)
*Study design*				
Cross-sectional	88	135,275	12,969	13.37 (10.68–16.30)
Prospective cohort	21	23,022	2756	17.90 (12.66–23.82)
Case–control	3	312	73	21.03 (3.67–46.99)
RCT	7	6108	995	20.15 (12.55–29.01)
*Publication year*				
Before 2000	14	3505	441	12.84 (9.24–16.93)
2001–2010	27	33,541	4365	16.12 (11.43–21.44)
2011–2022	78	127,671	11,987	14.53 (11.58–17.75)
*Risk of bias*				
Low	68	151,473	14,489	13.06 (10.26–16.15)
Moderate	51	13,244	2304	17.11 (13.64–20.87)

### Prevalence of MRSA colonization in ECCs

**Fig. 2:**
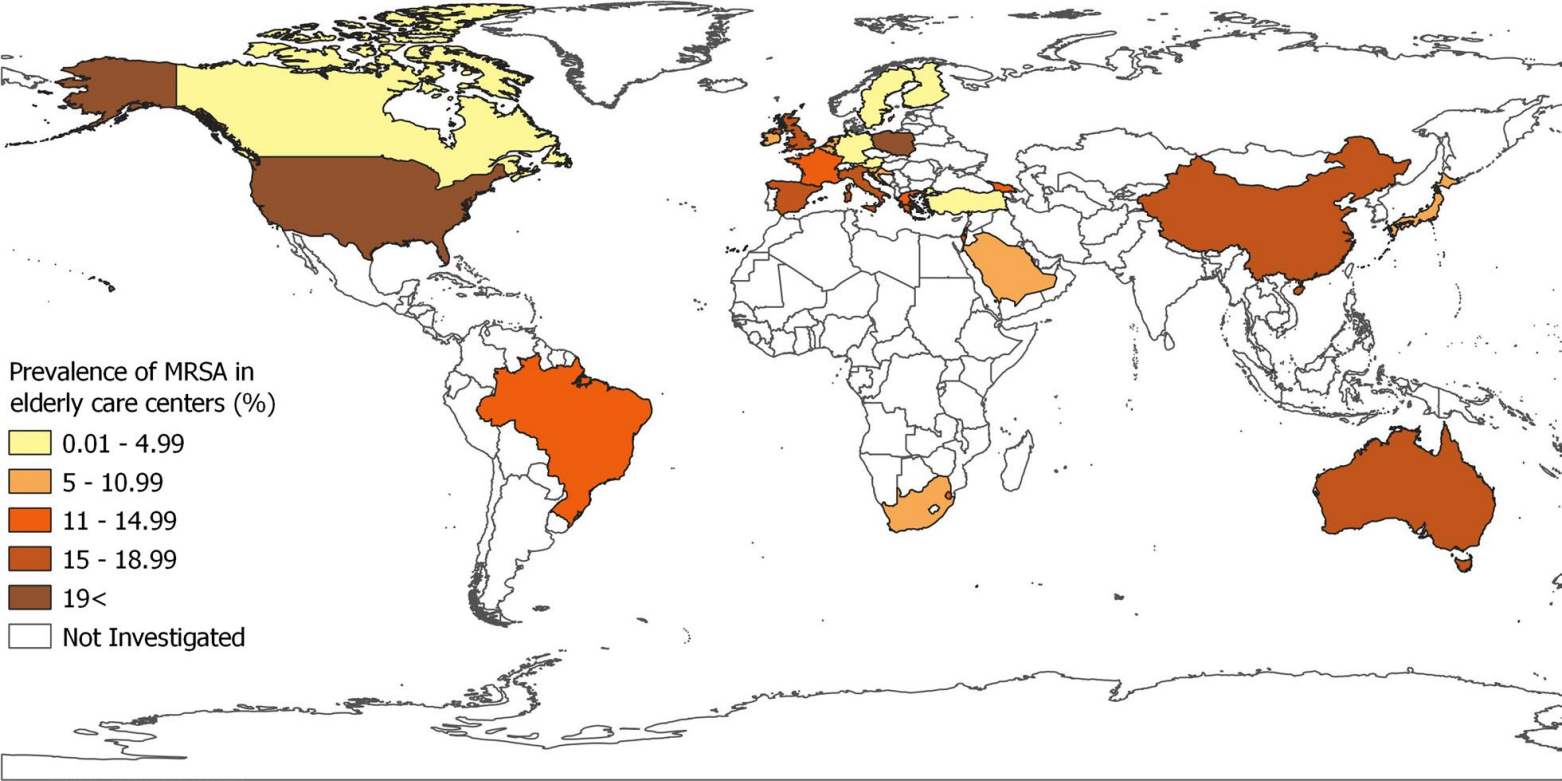
Worldwide distribution of MRSA colonization in ECCs

Table 2 presents the national and regional pooled prevalence of MRSA in residents of ECCs. The pooled global prevalence was 14.69% (95% CI 12.39–17.15%; 16,793/164,717) by using REM, with high heterogeneity across 119 studies (χ2 = 18,637.54, *P* < 0.001, *I*^2^ = 99.3%). According to WHO-defined regions, pooled prevalence rates (at 95% CI) were: 22.27% (15.56–29.79%) in the region of the Americas, 16.57% (11.70–22.10%) in the Western Pacific, 10.93% (8.56–13.55%) in Europe. Only one study was available for each of the Eastern Mediterranean and African regions, indicating prevalence rates of 8.55% (4.63–14.18%) and 9.04% (5.36–14.08%), respectively. For countries with two or more available studies, United States (23.78%), Singapore (22.72%), Poland (22.18%), United Kingdom (18.66%), China (18.07%), Italy (16.34%), Spain (15.45%), Israel (14.82%), France (13.89%) and Switzerland (13.15%) exhibited almost the highest prevalence rates (Fig. 2). Analyzing the data on the proportion of MRSA and MSSA in 26 studies using REM showed that 26% (18–36%) of all *S. aureus* isolates were MRSA (Additional file 1: Fig. S2A).

**Fig. 3:**
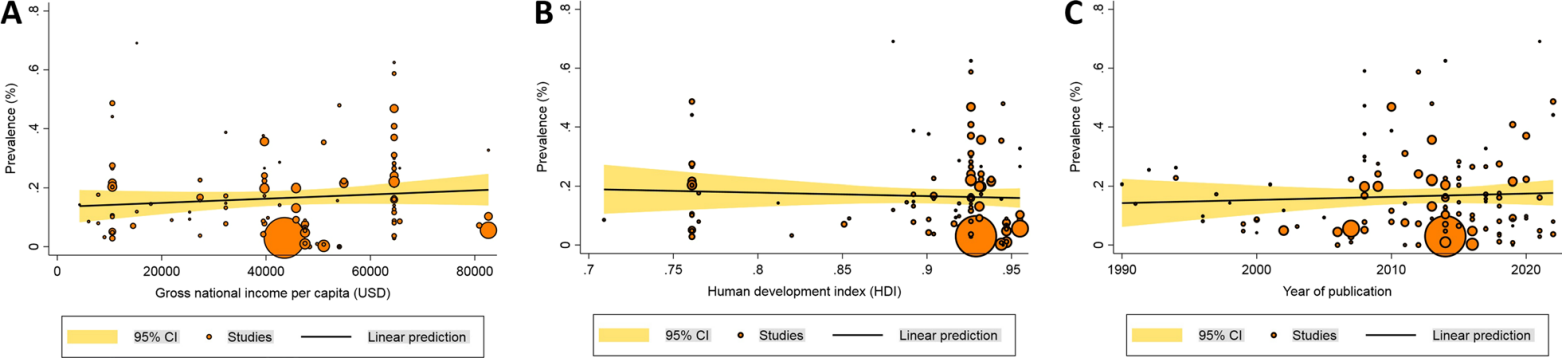
Meta-regression analyses of MRSA prevalence among elderly living in ECCs concerning **A** Country's gross national income per capita, **B** HDI level and **C** Publication year

Subgroup analyses of income and HDI levels yielded relatively similar results; prevalence rate for countries with upper-middle income and high HDI levels was 16.5%, and for countries with high income and very high HDI levels was 14.4% (see Table 2). Meta-regression analyses indicated a non-significant increasing trend in prevalence with higher income [coefficient (*C*) = 0.000001; *P* = 0.259], and HDI values (*C* = -0.117; *P* = 0.573) (Fig. 3A, B). According to the type of ECCs, pooled prevalence rates of MRSA were 16.29% (12.29–20.71%), 14.35% (11.50–17.44%), and 9.85% (3.62–17.93%) for the elderly living in long-term care facilities, nursing homes, and residential care homes, respectively. Considering study designs, the lowest and highest prevalence rates were observed in studies with cross-sectional (13.37%, 10.68–16.30%) and RCT (20.15%, 12.55–29.01%) designs, respectively. Studies published after year of 2000 showed non-significant increasing prevalence rates (*C* = 0.001; *P* = 0.547) (Table 2 and Fig. 3C). Studies with low risk of bias (13.06%, 10.26–16.15%) showed lower prevalence rates than those with moderate risk of bias (17.11%, 13.64–20.87%) (Table 2).

### Risk factors associated with MRSA colonization in residents of ECCs

The analyses of key determinants of MRSA colonization in elderly living in ECCs showed that MRSA is more colonized in males than females (PR = 1.55; 95% CI 1.47–1.64). With regard to other risk factors, we found that elderly with a previous history of MRSA infection (PR = 3.71; 95% CI 3.44–4.01), prior use of antibiotics (PR = 1.97; 95% CI 1.83–2.12), history of hospitalization within the previous year (PR = 1.32; 95% CI 1.20–1.45), those with any wound (PR = 2.38; 95% CI 2.23–2.55), those who have used urinary catheter (PR = 2.24; 95% CI 2.06–2.43), those who have used any medical device (PR = 1.78; 95% CI 1.66–1.91), and those with diabetes (PR = 1.55; 95% CI 1.43–1.67) were more likely to be colonized by MRSA than other patients (Table 3). Other risk factors such as antacid use and dementia were not significantly associated with an increased risk of MRSA colonization (*P*-value > 0.05). More information is presented in Table 3.

**Table 3:** Risk factors associated with MRSA colonization in elderly living in ECCs

Variable subgroup	Number of datasets	Number of elderlies screened (total)	Number of elderlies with MRSA	Pooled prevalence % (95% CI)	Prevalence ratio (95% CI)
*Gender*					
Male	32	9149	2164	18.41 (13.14–24.30)	1.55 (1.47–1.64)
Female	32	15,572	2387	15.18 (10.81–20.12)	1
*Prior antibiotics use*					
Yes	21	6746	1450	24.59 (17.82–32.03)	1.97 (1.83–2.12)
No	21	9492	1031	13.61 (9.67–18.09)	1
*Prior MRSA infection*					
Yes	11	1430	669	49.86 (35.87–63.86)	3.71 (3.44–4.01)
No	11	10,044	1264	17.76 (11.25–25.37)	1
*Hospitalization in past year*					
Yes	19	3149	559	21.34 (14.30–29.29)	1.32 (1.20–1.45)
No	18	6907	926	16.90 (10.99–23.76)	1
*Any wound*					
Yes	24	4003	1056	27.03 (19.62–35.10)	2.38 (2.23–2.55)
No	24	15,439	1706	12.41 (9.01–16.27)	1
*Urinary catheter*					
Yes	20	2949	661	19.59 (14.72–24.93)	2.24 (2.06–2.43)
No	20	15,656	1565	10.86 (7.44–14.83)	1
*Any device*					
Yes	16	5413	1274	30.09 (19.43–41.85)	1.78 (1.66–1.91)
No	16	9715	1279	16.07 (10.97–21.91)	1
*Diabetes*					
Yes	19	4990	870	17.11 (11.48–23.52)	1.55 (1.43–1.67)
No	19	13,734	1543	14.74 (9.69–20.60)	1
*Antacid use*					
Yes	3	1364	72	8.95 (1.98–19.36)	1.02 (0.76–1.38)
No	3	1966	101	5.01 (4.06–6.03)	1
*Dementia*					
Yes	5	1792	325	15.93 (7.93–25.97)	1.04 (0.93–1.17)
No	5	5243	994	15.08 (9.36–21.84)	1

## Discussion

MRSA infection continues to sustain as a major public health threat worldwide, especially in the elderly. In the present study, for the first time, we assembled data from all available studies (over 40 years) that had reported the prevalence of MRSA in ECCs. Among the key findings was the high pooled prevalence of MRSA colonization in the residents of ECCs (14.69%, 12.39–17.15%); which is over tenfold higher than the MRSA colonization rate among the general community (1.3%, 1.04–1.53%) [31]. Furthermore, the estimated colonization rate in our study is higher than those reported from other high-risk groups such as HIV + patients (7.0%, 5.0–9.0%) [32], hemodialysis patients (6.2%, 4.2–8.5%) [33], and patients admitted to intensive care units (7.0%, 5.8–8.3%) [34]. This highlights that elderly residents of ECCs are at very high risk for MRSA colonization, maybe due to cross-transmission between the elderlies in the crowded situation of ECCs or introduction of infection when admitting new elders from outside of ECCs (i.e., hospitals or community) [35]. Other possible explanations for this high prevalence of MRSA could be frailty, impaired immune system function, frequent hospitalization, and overuse of antibiotics [36, 37]. All the above-mentioned reasons underline the significance of recognizing contributing factors, and treating MRSA to reduce and prevent the spread of the disease.

Our results showed that the geographic distribution of MRSA colonization is heterogeneous, with the highest and lowest colonization rates reported in countries in regions of the Americas (22.27%) and Europe (10.93%), respectively. This finding is in accordance with previous meta-analysis studies in other high-risk populations [32–34]. Spatial variations of MRSA prevalence could be explained by differences in numerous demographic information among the countries studied and different ECCs in a country such as policies for prescription of antibiotics, various infection prevention programs, different education and training of staff and elderly for personal hygiene, different structure of health care systems, and facilities for MRSA diagnosis [32, 38–41]. Our study also highlighted a significant data gap in less developed countries, most strikingly in Latin America, Africa, Eastern Mediterranean, Central Asia, and South-East of Asia regions, where more data are required to obtain accurate estimates of the prevalence of MRSA colonization in elderly residents of ECCs. It should be noted that underdeveloped or developing countries may not have well-established elderly care systems or antibiotic stewardship programs. Due to the increasing number of ECCs in developing countries [42], these information gaps should be addressed through future representative epidemiological studies.

Our findings suggested different prevalence rates of MRSA colonization in nursing homes (14.35%), long-term care facilities (16.29%), and residential care homes (9.58%). The discrepancies in prevalence rates between different types of ECCs may stem from the differences in services rendered by each type of center. In nursing homes, patients usually receive daily or constant professional nursing care, and antibiotics can be prescribed with the consultation of a physician [43], therefore, antimicrobial overuse/abuse is prevalent [43]. In contrast, residential care centers are mostly restricted to personal care [44]. Despite monitoring schemes in hospitals for antibiotic use, records for the nursing homes environment are scarce [9]. Excessive use of antibiotics is one contributing factor to the increase in antibiotic resistance and the growth in the emergence of MDROs, which should be addressed in ECCs.

Our findings identified several risk factors for MRSA colonization in residents of ECCs, such as being male, prior antibiotics use, previous MRSA infection, hospitalization in the past 12 months, presence of any wound, urinary catheter, usage of any invasive medical device, and diabetes. Our findings are consistent with several studies about the MRSA prevalence in the community or high-risk populations such as HIV + and hemodialysis patients [2, 31–33, 45, 46]. Although there is no consensus on the role of gender in the prevalence of MRSA, it has been asserted that elderly males are more prone to the other predisposing factors of being infected with MRSA namely more complicated diabetes and wounds, frequent use of medical devices, and catheterization which may lead to a higher prevalence MRSA [47, 48]. Furthermore, frequent use of antibiotics is one of the main cause for the development of antimicrobial resistance in *staphylococci* and other bacteria [46, 49]. A previous MRSA infection could lead to re-infection secondary to a lack of proper eradication or stable colonization [45]. The constant presence of high-risk individuals in hospitals and routine antibiotics use justify the higher prevalence of MRSA in elders who were hospitalized in the last 12 months [34]. Medical devices make patients susceptible to MRSA through the mechanism of biofilm formation on the devices and subsequent detachment, which may contribute to bacteremia or sepsis [50]. A wide range of chronic diseases like diabetes renders the patients prone to MRSA given the state of immunosuppression and more complicated wounds [51, 52]. Similar to our findings, chronic illnesses, intravenous drug use, and contact with infected individuals are reported as risk factors for community-acquired MRSA [31]. These findings have implications for policymakers in identifying high-risk groups to reduce the disease burden by employing targeted interventions.

Our findings in this comprehensive systematic review and meta-analysis have implications for future research and clinical practices. However, this study has identified several shortcomings of the current data on MRSA colonization among the elderly in ECCs. First, our study contains reports from only 30 of ~ 200 countries globally; there were no country-level estimates for some countries, and no prevalence data were available from many countries. In addition, most of the available studies had a paucity of data on critical variables such as gender, age, ethnicity, and risk factors associated with MRSA colonization. Therefore, some non-significant results considering risk factors might be due to the low number of studies. For example, while among those who took antacids (as shown in Table 3) almost double MRSA (8.95%) has been estimated, the prevalence ratio was non-significant. Second, the studies included had different qualities and studies with lower qualities (17.11%) showed higher colonization rates than high quality studies (13.06%) which highlight the need for more robust surveys of MRSA colonization in residents of ECCs. Third, as expected in meta-analyses on prevalence studies [25, 29, 53–55], a substantial heterogeneity was found in our analyses that could not be explained by subgroup analyses. The sources of heterogeneity are likely related to study characteristics, including differences in geographical distribution, study design, sample size, diagnostic methods, leading to differences in the quality and performance of these methods. Moreover, this heterogeneity suggests that local risk factors and transmission routes of MRSA are varied in different geographical regions, countries, or even in individual ECCs.

## Conclusion

In conclusion, this study found that the prevalence of MRSA colonization in residents of ECCs is high across the world and varies by gender and geographic location. Moreover, elders with previous MRSA infection, hospitalization, antibiotics and diabetes, and those that used medical devices are more prone to MRSA colonization. This sheds significant light on the essence of targeted interventions and screening programs to locate infected people, control risk factors, and reduce the transmission of MRSA and other MDROs to elderlies living in ECCs. Given the aging trend of the populations, there is an urgent need for studies, particularly in developing and underdeveloped countries to better estimate the risk of disease. We recommend several interventions to reduce the MRSA burden in ECCS. For instance, reducing the consumptions of antibiotics (especially fluoroquinolones and third generation cephalosporins), enhanced barrier precautions, chlorhexidine bathing, routine MDROs surveillance, and isolation of MRSA-infected patients in single rooms. Additionally, educating and training the elderly and health workers in personal hygiene should be promoted.

## Supplementary Information

**Additional file 1.** Supplematary tables and figures.

## Data Availability

The data that support the findings of this study are available from the corresponding author upon reasonable request.
