# Supplementary material for: The global prevalence of methicillin-resistant Staphylococcus aureus colonization in residents of elderly care centers: a systematic review and meta-analysis
Source: Antimicrob Resist Infect Control. 2023 Jan 29;12:4. doi: 10.1186/s13756-023-01210-6 (PMC9884412; doi:10.1186/s13756-023-01210-6)
Supplement: Supplementary file 1 — Additional file 1. Supplematary tables and figures. [file 13756_2023_1210_MOESM1_ESM.docx]

**Table S1.** Main characteristics of all eligible studies reporting the prevalence of MRSA in elderly people living in ECCs

| **Author** | **Country** | **Income level** | **Type of ECCs** | **Type of study** | **Type of sample** | **Number of elderly people tested** | **Number of elderly people tested positive for MRSA** | **Quality score** |
| --- | --- | --- | --- | --- | --- | --- | --- | --- |
| **African region** |  |  |  |  |  |  |  |  |
| September et al. (2019) [1] | South Africa | UMI | RCHs | Cross sectional | Nasal, skin, inguinal, axillary | 152 | 13 | Moderate |
| **Eastern Mediterranean Region** |  |  |  |  |  |  |  |  |
| Albarrag et al. (2020) [2] | Saudi Arabia | High | NHs | Cross sectional | Nasal | 188 | 17 | Moderate |
| **Region of the Americas** |  |  |  |  |  |  |  |  |
| Gibson et al. (2022) [3] | United states | High | NHs | Prospective cohort | Nares, orophar-ynx, groin, perianal area | 625 | 23 | Moderate |
| Silva et al. (2022) [4] | Brazil | UMI | RCHs | Cross sectional | nasal vestibule, oropharynx, rectum | 226 | 18 | Low |
| Mody et al. (2021) [5] | United states | High | NHs | RCT | Nares, orophar-ynx, groin, perianal area | 245 | 7 | Low |
| Cassone et al. (2021) [6] | United states | High | NHs | Prospective cohort | Nasal, throat, inguinal, perineal | 651 | 105 | Low |
| McKinnell et al. (2020) [7] | United states | High | NHs | Cross sectional | Nasal, axillary, inguinal | 1400 | 518 | Low |
| McKinnell et al. (2019) [8] | United states | High | NHs | Cross sectional | Nasal, axillary, inguinal, perirectal | 1046 | 427 | Low |
| da Silveira et al. (2018) [9] | Brazil | UMI | NHs | Cross sectional | Nasal | 300 | 53 | Moderate |
| Heinze et al. (2018) [10] | United states | High | NHs | Prospective cohort | Nasal, throat, inguinal, wound | 508 | 41 | Low |
| Mody et al. (2018) [11] | United states | High | NHs | Prospective cohort | Nasal, throat, inguinal, wound, device | 651 | 105 | Low |
| McKinnell et al. (2016) [12] | United states | High | NHs | Cross sectional | Nasal, axillary, inguinal | 605 | 160 | Low |
| Roghmann et al. (2015) [13] | United states | High | NHs | Prospective cohort | Nasal, skin | 401 | 113 | Low |
| Datta et al. (2014) [14] | United states | High | NHs | Cross sectional | Nasal | 1661 | 266 | Low |
| Elemam et al. (2014) [15] | Canada | High | LTCFs | Cross sectional | Nasal | 64082 | 1920 | Low |
| Gibson et al. (2014) [16] | United states | High | NHs | RCT | Nasal, throat, urinary catheter, inguinal, perianal, wounds | 120 | 75 | Moderate |
| Mitchell et al. (2014) [17] | United states | High | NHs | Prospective cohort | Nasal, rectal | 360 | 46 | Low |
| Schora et al. (2014) [18] | United states | High | LTCFs | Cross sectional | Nasal | 673 | 112 | Low |
| Hudson et al. (2013) [19] | United states | High | NHs | Prospective cohort | Nasal | 3806 | 837 | Moderate |
| Murphy et al. (2012) [20] | United states | High | NHs | Cross sectional | Nasal | 2111 | 507 | Low |
| Stone et al. (2012) [21] | United states | High | LTCFs | Prospective cohort | Nasal | 412 | 242 | Low |
| Reynolds et al. (2011) [22] | United states | High | NHs | Cross sectional | Nasal | 1000 | 310 | Low |
| Bowler et al. (2010) [23] | United states | High | NHs | RCT | Nasal, wound, device, | 687 | 80 | Low |
| Li et al. (2010) [24] | United states (Hawai‘i) | High | NHs | Cross sectional | Wound, sputum, urine, blood | 2248 | 1053 | Low |
| Garazi et al. (2009) [25] | United states | High | NHs | Cross sectional | Nasal | 160 | 44 | Moderate |
| Furuno et al. (2008) [26] | United states | High | LTCFs | Cross sectional | Nasal, wound, rectal, sputum | 147 | 44 | Moderate |
| Mody et al. (2008) [27] | United states | High | NHs | Case-control | Nares, oropharynx, groin, perianal area, and wounds | 108 | 51 | Moderate |
| Stone et al. (2008) [28] | United states | High | LTCFs | Prospective cohort | Nasal | 83 | 49 | Moderate |
| Trick et al. (2001) [29] | United states | High | NHs | Cross sectional | Nasal, rectal, device, axillary, wound | 117 | 24 | Moderate |
| Smith et al. (2000) [30] | United states | High | LTCFs | Prospective cohort | Nasal, urine, wound | 442 | 39 | Low |
| Mulhausen et al. (1996) [31] | United states | High | NHs | Cross sectional | Nasal | 148 | 12 | Moderate |
| Owen et al. (1994) [32] | United states | High | NHs | Cross sectional | Nasal, oral, wound, device | 107 | 28 | Moderate |
| Terpenning et al. (1994) [33] | United states | High | LTCFs | Prospective cohort | Nasal, perineal, rectal, wound | 551 | 125 | Low |
| Murphy et al. (1992) [34] | United states | High | LTCFs | Cross sectional | Nasal, wound, sputum, stoma, urine | 118 | 30 | Moderate |
| Muder et al. (1991) [35] | United states | High | LTCFs | Cross sectional | Nasal | 164 | 23 | Moderate |
| Cederna et al. (1990) [36] | United states | High | NHs | RCT | Nasal | 102 | 21 | Moderate |
| **European Region** |  |  |  |  |  |  |  |  |
| Kasela et al. (2021) [37] | Poland | High | NHs | Cross sectional | Nasal, throat | 55 | 38 | Moderate |
| Moschou et al. (2020) [38] | Greece | High | NHs | Cross sectional | Nasal | 227 | 33 | Moderate |
| Drayß et al. (2019) [39] | Germany | High | NHs | Cross sectional | Nasal, throat | 46 | 3 | Moderate |
| Latour et al. (2019) [40] | Belgium | High | NHs | Cross sectional | Nasal, throat, perieal, wound | 1448 | 133 | Low |
| Olofsson et al. (2019) [41] | Sweden | High | NHs | Cross sectional | Nasal, throat, inguinal, wound, urine, rectal | 73 | 0 | Moderate |
| Van Dulm et al. (2019) [42] | Netherlands | High | LTCFs | Cross sectional | Nasal | 385 | 3 | Low |
| Galán et al. (2019) [43] | Spain | High | RCHs | Cross sectional | Nasal, axillary | 293 | 11 | Moderate |
| Nucleo et al. (2018) [44] | Italy | High | LTCFs | Cross sectional | Throat, rectal, inguinal | 340 | 45 | Low |
| Kohler et al. (2018) [45] | Switzerland | High | NHs | Prospective cohort |  | 9940 | 556 | Low |
| Kutsoylu et al. (2018) [46] | Turkey | High | NHs | Cross sectional | Nasal, axillary, rectal | 247 | 8 | Moderate |
| Kwetkat et al. (2018) [47] | Germany | High | NHs | Cross sectional | Nasal, throat | 529 | 6 | Low |
| Becker et al. (2017) [48] | Germany | High | NHs | Cross sectional | Nasal | 154 | 9 | Moderate |
| Giufrè et al. (2017) [49] | Italy | High | LTCFs | Cross sectional | Nasal, axillary | 487 | 84 | Low |
| Hequet et al. (2017) [50] | Switzerland | High | NHs | RCT | Nasal, throat, inguinal | 104 | 34 | Moderate |
| March et al. (2017) [51] | Italy | High | LTCFs | Cross sectional | Throat, rectal, inguinal | 115 | 17 | Moderate |
| Peters et al. (2017) [52] | Germany | High | NHs | Cross sectional | Nasal, wound | 422 | 23 | Low |
| Reynaga et al. (2017) [53] | Spain | High | NHs | Prospective cohort | Nasal | 204 | 32 | Moderate |
| Harrison et al. (2016) [54] | United Kingdom | High | LTCFs | Prospective cohort | Nasal | 64 | 17 | Moderate |
| Nillius et al. (2016) [55] | Germany | High | LTCFs | Cross sectional | Nasal, throat | 2858 | 136 | Low |
| Rondeau et al. (2016) [56] | France | High | LTCFs | Cross sectional | Blood | 921 | 39 | Low |
| Rondeau et al. (2016) [56] | France | High | NHs | Cross sectional | Blood | 470 | 41 | Low |
| Verhoef et al. (2016) [57] | Netherlands | High | LTCFs | Cross sectional | Nasal, urine | 4763 | 13 | Low |
| Hogardt et al. (2015) [58] | Germany | High | LTCFs | Cross sectional | Nasal, throat, perianal | 690 | 45 | Low |
| Bellini et al. (2015) [59] | Switzerland | High | NHs | RCT | Nasal, inguinal, wound | 2084 | 215 | Low |
| Ludden et al. (2015) [60] | Ireland | High | LTCFs | Cross sectional | Nasal | 64 | 17 | Moderate |
| Vendrell et al. (2015) [61] | Spain | High | LTCFs | Cross sectional | Nasal, wound | 413 | 93 | Low |
| Barrufet et al. (2014) [62] | Spain | High | LTCFs | Cross sectional | Nasal, wound | 413 | 93 | Low |
| Budimir et al. (2014) [63] | Croatia | High | NHs | Cross sectional | Nasal, wound, urine | 877 | 62 | Low |
| March et al. (2014) [64] | Italy | High | LTCFs | Cross sectional | Nasal, orothroat, rectal, urine, inguinal | 106 | 14 | Moderate |
| Romaniszyn et al. (2014) [65] | Poland | High | LTCFs | Cross sectional | Nasal, wound | 193 | 23 | Moderate |
| Ruscher et al. (2014) [66] | Germany | High | LTCFs | Cross sectional | Inguinal | 402 | 19 | Low |
| Stark et al. (2014) [67] | Sweden | High | NHs | Prospective cohort | Nasal, throat, wound, inguinal | 290 | 139 | Moderate |
| Gruber et al. (2013) [68] | Germany | High | NHs | Cross sectional | Nasal,throat, rectal, wounds | 178 | 16 | Moderate |
| Horner et al. (2013) [69] | United Kingdom | High | NHs | Cross sectional | Nasal | 2492 | 888 | Low |
| Jans et al. (2013) [70] | Belgium | High | NHs | Cross sectional | Nasal, wound, perineal, throat | 2789 | 366 | Low |
| Mossong et al. (2013)[71] | Luxembourg | High | LTCFs | Cross sectional | Nasal, throat | 954 | 69 | Low |
| van der Donk et al. (2013) [72] | Netherlands | High | NHs | Cross sectional | Nasal | 693 | 245 | Low |
| Andersson et al. (2012) [73] | Sweden | High | NHs | Cross sectional | Nasal, throat, wound, Urainary catheter | 560 | 0 | Low |
| Schoevaerdts et al. (2012) [74] | Belgium | High | LTCFs | Prospective cohort | Nasal, throat,inguinal, axillary | 320 | 24 | Moderate |
| Olofsson et al. (2012) [75] | Sweden | High | NHs | Cross sectional | Nasal, throat, inguinal, wound | 199 | 0 | Moderate |
| Pfingsten-Würzburg et al. (2011) [76] | Germany | High | NHs | Cross sectional | Nasal, wound | 1827 | 139 | Moderate |
| Schwaber et al. (2011) [77] | Israel | High | LTCFs | Cross sectional | Nasal | 191 | 27 | Moderate |
| Lasseter et al. (2010) [78] | United Kingdom | High | NHs | Cross sectional | Nasal | 748 | 59 | low |
| March et al. (2010) [79] | Italy | High | LTCFs | Cross sectional | Throat, rectal, inguinal | 111 | 43 | Moderate |
| Baldwin et al. (2009) [80] | United Kingdom | High | NHs | Cross sectional | Nasal, wound | 1111 | 267 | Low |
| Denis et al. (2009) [81] | Belgium | High | NHs | Cross sectional | Nasal, throat, wound, Urainary meatus | 2953 | 587 | Low |
| Brugnaro et al. (2009) [82] | Italy | High | LTCFs | Cross sectional | Nasal | 551 | 43 | Low |
| Eveillard et al. (2008) [83] | France | High | LTCFs | Cross sectional | Nasal, rectal, urine, wound | 109 | 41 | Moderate |
| Manzur et al. (2008) [84] | Spain | High | LTCFs | Cross sectional | Nasal, wound | 1377 | 231 | Low |
| Pop-Vicas et al. (2008) [85] | Israel | High | LTCFs | Cross sectional | Nasal, rectal | 84 | 24 | Moderate |
| Smith et al. (2008) [86] | United Kingdom | High | NHs | Cross sectional | Nasal | 3037 | 601 | Low |
| Barr et al. (2007) [87] | United Kingdom | High | NHs | Cross sectional | Nasal | 715 | 159 | Low |
| Kerttula et al. (2007) [88] | Finland | High | NHs | Cross sectional | Nasal, throat, perieal | 213 | 2 | Moderate |
| Daeschlein et al. (2006) [89] | Austria | High | NHs | Cross sectional | Nasal | 500 | 0 | Low |
| Suetens et al. (2006) [90] | Belgium | High | NHs | Prospective cohort | Nasal, perineal, urine, wound | 2694 | 120 | Low |
| Cretnik et al. (2005) [91] | Slovenia | High | LTCFs | Cross sectional | Nasal, wound | 107 | 10 | Moderate |
| Vovko et al. (2005) [92] | Slovenia | High | LTCFs | Case-control | Nasal, wound, axillary, inguinal | 102 | 12 | Moderate |
| Mendelson et al. (2003) [93] | Israel | High | LTCFs | Cross sectional | Nasal, throat | 270 | 17 | Moderate |
| Baum et al. (2002) [94] | Germany | High | NHs | Cross sectional | Nasal, wound | 3751 | 36 | Low |
| Hoefnagels-Schuermans et al. (2002) [95] | Belgium | High | NHs | Cross sectional | Nasal, perineal | 2857 | 141 | Low |
| O'Sullivan et al. (2000) [96] | Ireland | High | NHs | Cross sectional | Nasal, throat, hairline, axillary, perineal, wound | 754 | 65 | Low |
| Cox et al. (1999) [97] | United Kingdom | High | NHs | Cross sectional | Nasal, throat, wound, perineal | 275 | 13 | Moderate |
| Niclaes et al. (1999) [98] | Belgium | High | NHs | Prospective cohort | Nasal, throat, perieal | 447 | 32 | Low |
| Rahimi et al. (1998) [99] | Georgia | UMI | NHs | Prospective cohort | Nasal | 56 | 8 | Moderate |
| Fraise et al. (1997) [100] | United Kingdom | High | NHs | Cross sectional | Nasal, wound | 191 | 33 | Moderate |
| **Western Pacific Region** |  |  |  |  |  |  |  |  |
| Wong et al. (2022) [101] | China | UMI | LTCFs | Cross sectional | Nasal, axillary, groin, rectal | 781 | 380 | Low |
| Huang et al. (2022) [102] | China (Taiwan) | UMI | NHs and LTCFs | Cross sectional | Nasal | 127 | 56 | Moderate |
| He et al. (2021) [103] | China | UMI | NHs | Cross sectional | Nasal, axillary, skin | 496 | 24 | Low |
| Chow et al. (2020) [104] | Singapore | High | LTCFs | Cross sectional | Nasal, axillary, inguinal | 1157 | 257 | Low |
| Sasahara et al. (2020) [105] | Japan | High | LTCFs | Prospective cohort | Nasal | 204 | 20 | Moderate |
| Htun et al. (2019) [106] | Singapore | High | LTCFs | Cross sectional | Nasal, axillary, inguinal | 2456 | 528 | Low |
| Chen et al. (2018) [107] | China | UMI | RCHs | Cross sectional | Nasal, axillary, rectal | 1028 | 282 | Low |
| Lee et al. (2017) [108] | China (Taiwan) | UMI | LTCFs | Prospective cohort | Nasal, rectal | 313 | 83 | Low |
| Gu et al. (2016) [109] | China | UMI | NHs | Cross sectional | Nasal, axillary | 443 | 45 | Low |
| Tsao et al. (2015) [110] | China (Taiwan) | UMI | NHs | Cross sectional | Nasal | 360 | 73 | Moderate |
| Zhang et al. (2015) [111] | China | UMI | NHs | Cross sectional | Nasal, axillary, skin | 491 | 52 | Low |
| Chuang et al. (2015) [112] | China | UMI | RCHs | RCT | Nasal, wound, Urainary catheter | 2766 | 563 | Low |
| Lim et al. (2014) [113] | Australia | High | LTCFs | Cross sectional | Nasal | 115 | 18 | Moderate |
| Cheng et al. (2013) [114] | China (Hong Kong) | UMI | NHs | Cross sectional | Nasal | 2020 | 436 | Low |
| Ho et al. (2008) [115] | China | UMI | RCHs | Cross sectional | Nasal, wound | 1563 | 80 | Low |
| Ho et al. (2007) [116] | China | UMI | RCHs | Cross sectional | Nasal, wound | 949 | 27 | Low |
| Ishihara et al. (2000) [117] | Japan | High | NHs | Cross sectional | Oral | 48 | 2 | Moderate |
| washio et al. (1996) [118] | Japan | High | NHs | Case-control | Sputum, urine, pus | 102 | 10 | Moderate |

**Abbreviations: RCHs,** Residential Care Homes**; LTCFs**, Long-term care facilities; **NHs**, Nursing homes; **USA,** United States of America; **UK**, United Kingdom, **UMI**, upper middle income; **RCT**, Randomized Controlled Trial


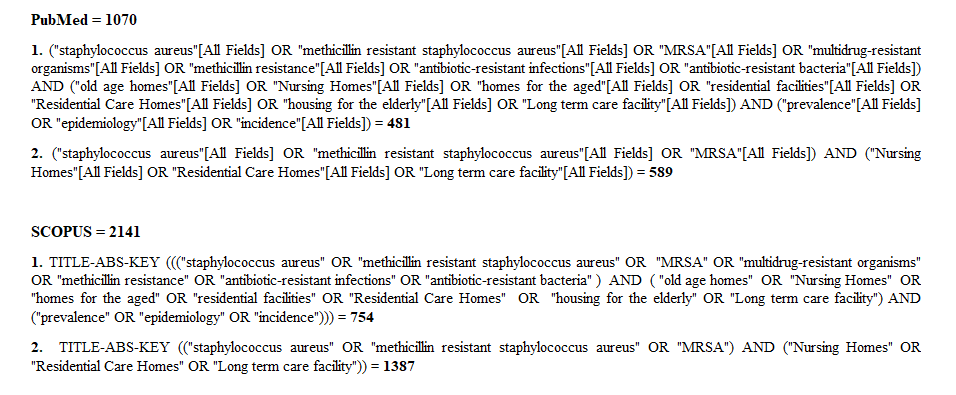


**Fig. S1. Search strategy**


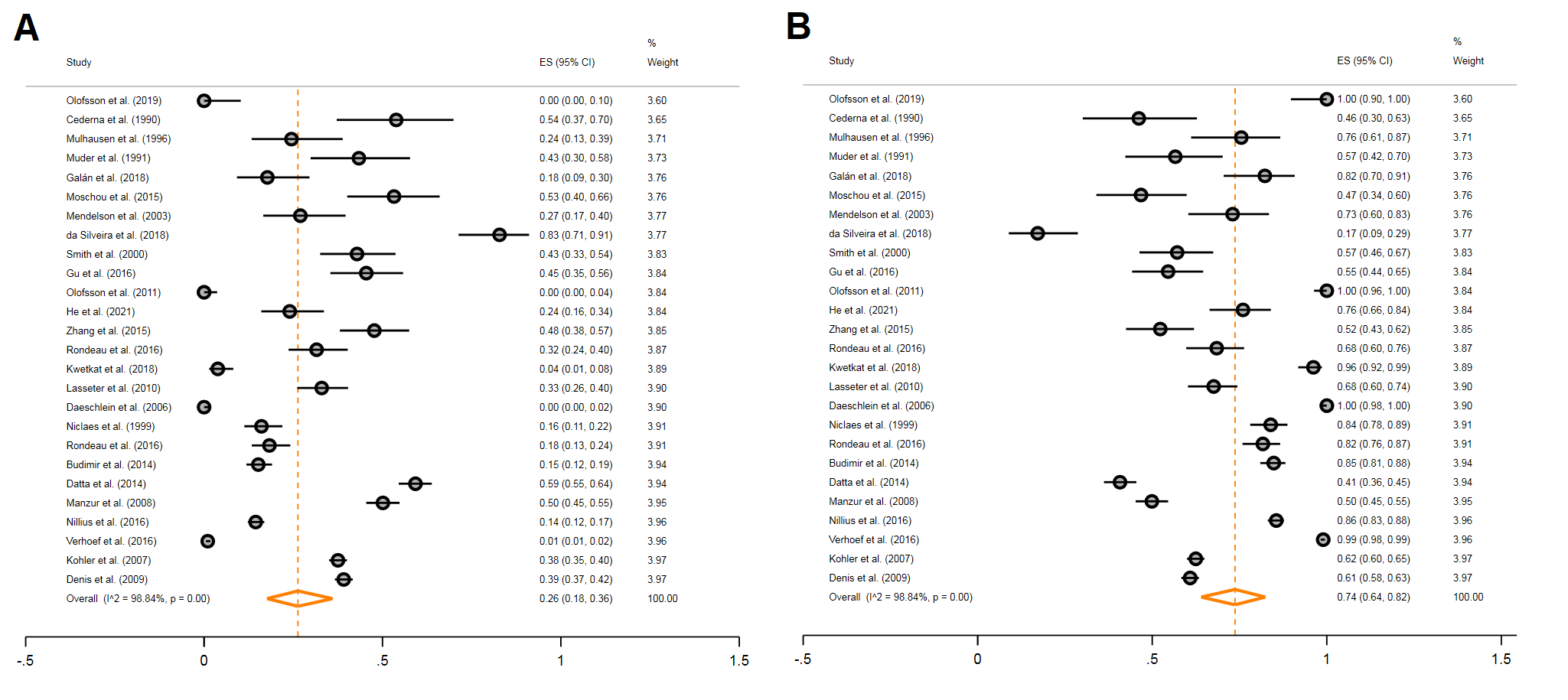


**Figure S2.** Proportion of MRSA (A) and MSSA (B) of *Staphylococcus aureus* isolates in residents of ECCs.

**Reference**

1. September J, Geffen L, Manning K, Naicker P, Faro C, Mendelson M, Wasserman S: **Colonisation with pathogenic drug-resistant bacteria and Clostridioides difficile among residents of residential care facilities in Cape Town, South Africa: A cross-sectional prevalence study**. *Antimicrob Resist Infect Control* 2019, **8**(1).

2. Albarrag A, Shami A, Almutairi A, Alsudairi S, Aldakeel S, Al-Amodi A: **Prevalence and Molecular Genetics of Methicillin-Resistant Staphylococcus aureus Colonization in Nursing Homes in Saudi Arabia**. *Can J Infect Dis Med Microbiol* 2020, **2020**.

3. Gibson KE, Mills JP, Mantey JA, Lansing BJ, Cassone M, Mody L: **Multidrug-resistant organism (MDRO) contamination of privacy curtains in nursing homes**. *Infection Control & Hospital Epidemiology* 2022, **43**(5):666-668.

4. Silva LP, Fortaleza CMCB, Teixeira NB, Silva LTP, de Angelis CD, Ribeiro de Souza da Cunha MdL: **Molecular Epidemiology of Staphylococcus aureus and MRSA in Bedridden Patients and Residents of Long-Term Care Facilities**. *Antibiotics* 2022, **11**(11):1526.

5. Mody L, Gontjes KJ, Cassone M, Gibson KE, Lansing BJ, Mantey J, Kabeto M, Galecki A, Min L: **Effectiveness of a multicomponent intervention to reduce multidrug-resistant organisms in nursing homes: a cluster randomized clinical trial**. *JAMA Network Open* 2021, **4**(7):e2116555-e2116555.

6. Cassone M, Linder M, Shin CJ, Mantey J, Gibson K, Lansing B, Mody L: **Not too close! impact of roommate status on MRSA and VRE colonization and contamination in Nursing Homes**. *Antimicrob Resist Infect Control* 2021, **10**(1).

7. McKinnell JA, Miller LG, Singh RD, Gussin G, Kleinman K, Mendez J, Laurner B, Catuna TD, Heim L, Saavedra R *et al*: **High Prevalence of Multidrug-Resistant Organism Colonization in 28 Nursing Homes: An “Iceberg Effect”**. *J Am Med Dir Assoc* 2020, **21**(12):1937-1943.e1932.

8. McKinnell JA, Singh RD, Miller LG, Kleinman K, Gussin G, He J, Saavedra R, Dutciuc TD, Estevez M, Chang J *et al*: **The SHIELD Orange County Project: Multidrug-resistant Organism Prevalence in 21 Nursing Homes and Long-term Acute Care Facilities in Southern California**. *Clin Infect Dis* 2019, **69**(9):1566-1573.

9. da Silveira M, Cunha MLRS, de Souza CSM, Correa AAF, Fortaleza CMCB: **Nasal colonization with methicillin-resistant Staphylococcus aureus among elderly living in nursing homes in Brazil: Risk factors and molecular epidemiology**. *Ann clin microbiol* 2018, **17**(1).

10. Heinze K, Kabeto M, Martin ET, Cassone M, Hicks L, Mody L: **Predictors of methicillin-resistant Staphylococcus aureus and vancomycin-resistant enterococci co-colonization among nursing facility patients**. *Am J Infect Control* 2019, **47**(4):415-420.

11. Mody L, Foxman B, Bradley S, McNamara S, Lansing B, Gibson K, Cassone M, Armbruster C, Mantey J, Min L: **Longitudinal assessment of multidrug-resistant organisms in newly admitted nursing facility patients: Implications for an evolving population**. *Clin Infect Dis* 2018, **67**(6):837-844.

12. McKinnell JA, Miller LG, Singh R, Kleinman K, Peterson EM, Evans KD, Dutciuc TD, Heim L, Gombosev A, Estevez M *et al*: **Prevalence of and Factors Associated with Multidrug Resistant Organism (MDRO) Colonization in 3 Nursing Homes**. *Infect Control Hosp Epidemiol* 2016, **37**(12):1485-1488.

13. Roghmann MC, Johnson JK, Sorkin JD, Langenberg P, Lydecker A, Sorace B, Levy L, Mody L: **Transmission of Methicillin-Resistant Staphylococcus aureus (MRSA) to Healthcare Worker Gowns and Gloves During Care of Nursing Home Residents**. *Infect Control Hosp Epidemiol* 2015, **36**(9):1050-1057.

14. Datta R, Quan V, Kim D, Peterson EM, Reynolds C, Meyers H, Cheung M, Huang SS: **Protective effect of methicillin-susceptible Staphylococcus aureus carriage against methicillin-resistant S. aureus acquisition in nursing homes: A prospective cross-sectional study**. *Infect Control Hosp Epidemiol* 2014, **35**(10):1257-1262.

15. El Emam K, Arbuckle L, Essex A, Samet S, Eze B, Middleton G, Buckeridge D, Jonker E, Moher E, Earle C: **Secure surveillance of antimicrobial resistant organism colonization or infection in ontario long term care homes**. *PLoS ONE* 2014, **9**(4).

16. Gibson KE, McNamara SE, Cassone M, Perri MB, Zervos M, Mody L: **Methicillin-resistant Staphylococcus aureus: site of acquisition and strain variation in high-risk nursing home residents with indwelling devices**. *Infect Control Hosp Epidemiol* 2014, **35**(12):1458-1465.

17. Mitchell SL, Shaffer ML, Loeb MB, Givens JL, Habtemariam D, Kiely DK, D'Agata E: **Infection management and multidrug-resistant organisms in nursing home residents with advanced dementia**. *JAMA Intern Med* 2014, **174**(10):1660-1667.

18. Schora DM, Boehm S, Das S, Patel PA, O'Brien J, Hines C, Burdsall D, Beaumont J, Peterson K, Fausone M *et al*: **Impact of Detection, Education, Research and Decolonization without Isolation in Long-term care (DERAIL) on methicillin-resistant Staphylococcus aureus colonization and transmission at 3 long-term care facilities**. *Am J Infect Control* 2014, **42**(10):S269-S273.

19. Hudson LO, Reynolds C, Spratt BG, Enright MC, Quan V, Kim D, Hannah P, Mikhail L, Alexander R, Moore DF *et al*: **Diversity of methicillin-resistant staphylococcus aureus strains isolated from residents of 26 nursing homes in orange county, california**. *J Clin Microbiol* 2013, **51**(11):3788-3795.

20. Murphy CR, Quan V, Kim D, Peterson E, Whealon M, Tan G, Evans K, Meyers H, Cheung M, Lee BY *et al*: **Nursing home characteristics associated with methicillin-resistant Staphylococcus aureus (MRSA) Burden and Transmission**. *BMC Infect Dis* 2012, **12**.

21. Stone ND, Lewis DR, Johnson Jr TM, Hartney T, Chandler D, Byrd-Sellers J, McGowan Jr JE, Tenover FC, Jernigan JA, Gaynes RP: **Methicillin-resistant Staphylococcus aureus (MRSA) nasal carriage in residents of veterans affairs long-term care facilities: Role of antimicrobial exposure and MRSA acquisition**. *Infect Control Hosp Epidemiol* 2012, **33**(6):551-557.

22. Reynolds C, Quan V, Kim D, Peterson E, Dunn J, Whealon M, Terpstra L, Meyers H, Cheung M, Lee B *et al*: **Methicillin-resistant Staphylococcus aureus (MRSA) carriage in 10 nursing homes in Orange County, California**. *Infect Control Hosp Epidemiol* 2011, **32**(1):91-93.

23. Bowler WA, Bresnahan J, Bradfish A, Fernandez C: **An integrated approach to methicillin-resistant staphylococcus aureus control in a rural, regional-referral healthcare setting**. *Infect Control Hosp Epidemiol* 2010, **31**(3):269-275.

24. Li F, Arnsberger P, Miller FD: **Profile of methicillin-resistant Staphylococcus aureus among nursing home residents in Hawai'i**. *Hawaii Med J* 2010, **69**(5):126-129.

25. Garazi M, Edwards B, Caccavale D, Auerbach C, Wolf-Klein G: **Nursing Homes as Reservoirs of MRSA: Myth or Reality?** *J Am Med Dir Assoc* 2009, **10**(6):414-418.

26. Furuno JP, Hebden JN, Standiford HC, Perencevich EN, Miller RR, Moore AC, Strauss SM, Harris AD: **Prevalence of methicillin-resistant Staphylococcus aureus and Acinetobacter baumannii in a long-term acute care facility**. *Am J Infect Control* 2008, **36**(7):468-471.

27. Mody L, Kauffman CA, Donabedian S, Zervos M, Bradley SF: **Epidemiology of Staphylococcus aureus colonization in nursing home residents**. *Clin Infect Dis* 2008, **46**(9):1368-1373.

28. Stone ND, Lewis DR, Lowery HK, Darrow LA, Kroll CM, Gaynes RP, Jernigan JA, McGowan Jr JE, Tenover FC, Richards Jr CL: **Importance of bacterial burden among methicillin-resistant Staphylococcus aureus carriers in a long-term care facility**. *Infect Control Hosp Epidemiol* 2008, **29**(2):143-148.

29. Trick WE, Weinstein RA, Demarais PL, Kuehnert MJ, Tomaska W, Nathan C, Rice TW, McAllister SK, Carson LA, Jarvis WR: **Colonization of skilled-care facility residents with antimicrobial-resistant pathogens**. *J Am Geriatr Soc* 2001, **49**(3):270-276.

30. Smith PW, Seip CW, Schaefer SC, Bell-Dixon C: **Microbiologic survey of long-term care facilities**. *Am J Infect Control* 2000, **28**(1):8-13.

31. Mulhausen PL, Harrell LJ, Weinberger M, Kochersberger GG, Feussner JR: **Contrasting methicillin-resistant Staphylococcus aureus colonization in Veterans Affairs and community nursing homes**. *Am J Med* 1996, **100**(1):24-31.

32. Owen MK: **Prevalence of oral methicillin-resistant Staphylococcus aureus in an institutionalized veterans population**. *Spec Care Dentist* 1994, **14**(2):75-79.

33. Terpenning MS, Bradley SF, Wan JY, Chenoweth CE, Jorgensen KA, Kauffman CA: **Colonization and infection with antibiotic-resistant bacteria in a long-term care facility**. *J Am Geriatr Soc* 1994, **42**(10):1062-1069.

34. Murphy S, Denman S, Bennett RG, Greenough WB, III, Lindsay J, Zelesnick LB: **Methicillin‐Resistant Staphylococcus aureus Colonization in a Long‐Term‐Care Facility**. *J Am Geriatr Soc* 1992, **40**(3):213-217.

35. Muder RR, Wagener MM, Yu VL, Cherubin CE, Antar S, Jurkovic J: **Methicillin-resistant Staphylococcus aureus in nursing homes [4]**. *Ann Intern Med* 1992, **116**(3):267-268.

36. Cederna JE, Terpenning MS, Ensberg M, Bradley SF, Kauffman CA: **Staphylococcus aureus nasal colonization in a nursing home: eradication with mupirocin**. *Infect Control Hosp Epidemiol* 1990, **11**(1):13-16.

37. Kasela M, Grzegorczyk A, Nowakowicz-Dębek B, Malm A: **The prevalence of virulence determinants and antibiotic resistance patterns in methicillin—resistant staphylococcus aureus in a nursing home in Poland**. *Pathogens* 2021, **10**(4).

38. Moschou A, Maraki S, Giormezis N, Moraitaki H, Stafylaki D, Militsopoulou M, Spiliopoulou I, Papadakis JA, Samonis G, Kofteridis DP: **Prevalence and molecular epidemiology of Staphylococcus aureus nasal colonization in four nursing home residents in Crete, Greece**. *J Infect Chemother* 2020, **26**(2):199-204.

39. Drayß M, Claus H, Hubert K, Thiel K, Berger A, Sing A, Linden MV, Vogel U, Lâm TT: **Asymptomatic carriage of Neisseria meningitidis, Haemophilus influenzae, Streptococcus pneumoniae, Group A Streptococcus and Staphylococcus aureus among adults aged 65 years and older**. *PLoS One* 2019, **14**(2):e0212052.

40. Latour K, Huang TD, Jans B, Berhin C, Bogaerts P, Noel A, Nonhoff C, Dodémont M, Denis O, Ieven M *et al*: **Prevalence of multidrug-resistant organisms in nursing homes in Belgium in 2015**. *PLoS ONE* 2019, **14**(3).

41. Olofsson M, Matussek A, Ehricht R, Lindgren PE, Östgren CJ: **Differences in molecular epidemiology of Staphylococcus aureus and Escherichia coli in nursing home residents and people in unassisted living situations**. *J Hosp Infect* 2019, **101**(1):76-83.

42. Van Dulm E, Tholen ATR, Pettersson A, Van Rooijen MS, Willemsen I, Molenaar P, Damen M, Gruteke P, Oostvogel P, Kuijper EJ *et al*: **High prevalence of multidrug resistant Enterobacteriaceae among residents of long term care facilities in Amsterdam, the Netherlands**. *PLoS ONE* 2019, **14**(9).

43. Galán-Sánchez F, Pérez-Eslava M, Machuca J, Trujillo-Soto T, Arca-Suarez J, Rodríguez-Iglesias M: **Staphylococcus aureus carriage in older populations in community residential care homes: Prevalence and molecular characterization of MRSA isolates**. *Enferm Infecc Microbiol Clin* 2019, **37**(3):172-175.

44. Nucleo E, Caltagirone M, Marchetti VM, D'Angelo R, Fogato E, Confalonieri M, Reboli C, March A, Sleghel F, Soelva G *et al*: **Colonization of long-term care facility residents in three Italian Provinces by multidrug-resistant bacteria**. *Antimicrob Resist Infect Control* 2018, **7**:33.

45. Kohler P, Fulchini R, Albrich WC, Egli A, Balmelli C, Harbarth S, Héquet D, Kahlert CR, Kuster SP, Petignat C *et al*: **Antibiotic resistance in Swiss nursing homes: Analysis of National Surveillance Data over an 11-year period between 2007 and 2017**. *Antimicrob Resist Infect Control* 2018, **7**(1).

46. EREN KUTSOYLU OÖ, ABDULLAYEVA M, TEKİN N, YAPAR N, AVKAN OĞUZ V: **Prevalence of multidrug-resistant bacterial colonization and risk factors in geriatric nursing home residents**. *Türk Geriatri Dergisi* 2018, **21**(1):41-48.

47. Kwetkat A, Pfister W, Pansow D, Pletz MW, Sieber CC, Hoyer H: **Naso- and oropharyngeal bacterial carriage in nursing home residents: Impact of multimorbidity and functional impairment**. *PLoS ONE* 2018, **13**(1).

48. Becker J, Diel R: **Screening for Methicillin-resistant Staphylococcus aureus in a residence home for elderly in Germany**. *J Occup Med Toxicol* 2017, **12**(1).

49. Giufrè M, Ricchizzi E, Accogli M, Barbanti F, Monaco M, Pimentel de Araujo F, Farina C, Fazii P, Mattei R, Sarti M *et al*: **Colonization by multidrug-resistant organisms in long-term care facilities in Italy: a point-prevalence study**. *Clin Microbiol Infect* 2017, **23**(12):961-967.

50. Héquet D, Rousson V, Blanc DS, Büla C, Qalla-Widmer L, Masserey E, Zanetti G, Petignat C: **Universal screening and decolonization for control of MRSA in nursing homes: follow-up of a cluster randomized controlled trial**. *J Hosp Infect* 2017, **96**(1):69-71.

51. March A, Aschbacher R, Sleghel F, Soelva G, Kaczor M, Migliavacca R, Piazza A, Mattioni Marchetti V, Pagani L, Scalzo K *et al*: **Colonization of residents and staff of an Italian long-term care facility and an adjacent acute care hospital geriatric unit by multidrug-resistant bacteria**. *New Microbiol* 2017, **40**(4):258-263.

52. Peters C, Dulon M, Kleinmüller O, Nienhaus A, Schablon A: **MRSA prevalence and risk factors among health personnel and residents in nursing homes in Hamburg, Germany - A cross-sectional study**. *PLoS ONE* 2017, **12**(1).

53. Reynaga E, Torres C, Garcia-Nuñez M, Navarro M, Vilamala A, Puigoriol E, Lucchetti GE, Sabrià M: **Clinical impact and prevalence of MRSA CC398 and differences between MRSA-TetR and MRSA-TetS in an area of Spain with a high density of pig farming: a prospective cohort study**. *Clin Microbiol Infect* 2017, **23**(9):678.e671-678.e674.

54. Harrison EM, Ludden C, Brodrick HJ, Blane B, Brennan G, Morris D, Coll F, Reuter S, Brown NM, Holmes MA *et al*: **Transmission of methicillin-resistant Staphylococcus aureus in long-term care facilities and their related healthcare networks**. *Genome Med* 2016, **8**(1):102.

55. Nillius D, Von Müller L, Wagenpfeil S, Klein R, Herrmann M: **Methicillin-resistant staphylococcus aureus in Saarland, Germany: The long-term care facility study**. *PLoS ONE* 2016, **11**(4).

56. Rondeau C, Chevet G, Blanc DS, Gbaguidi-Haore H, Decalonne M, Dos Santos S, Quentin R, van der Mee-Marquet N, Amirault P, Guimard Y *et al*: **Current molecular epidemiology of methicillin-resistant staphylococcus aureus in elderly French people: Troublesome clones on the horizon**. *Front Microbiol* 2016, **7**(JAN).

57. Verhoef L, Roukens M, de Greeff S, Meessen N, Natsch S, Stobberingh E: **Carriage of antimicrobial-resistant commensal bacteria in Dutch long-term-care facilities**. *J Antimicrob Chemother* 2016, **71**(9):2586-2592.

58. Hogardt M, Proba P, Mischler D, Cuny C, Kempf VA, Heudorf U: **Current prevalence of multidrug-resistant organisms in long-term care facilities in the Rhine-Main district, Germany, 2013**. *Euro Surveill* 2015, **20**(26).

59. Bellini C, Petignat C, Masserey E, Büla C, Burnand B, Rousson V, Blanc DS, Zanetti G: **Universal Screening and Decolonization for Control of MRSA in Nursing Homes: A Cluster Randomized Controlled Study**. *Infect Control Hosp Epidemiol* 2015, **36**(4):401-408.

60. Ludden C, Cormican M, Vellinga A, Johnson JR, Austin B, Morris D: **Colonisation with ESBL-producing and carbapenemase-producing Enterobacteriaceae, vancomycin-resistant enterococci, and meticillin-resistant Staphylococcus aureus in a long-term care facility over one year**. *BMC Infect Dis* 2015, **15**:168.

61. !!! INVALID CITATION !!! [60].

62. Barrufet MP, Vendrell E, Force L, Sauca G, Rodríguez S, Martínez E, Palomera E, Serra-Prat M, Capdevila JA, Cornudella J *et al*: **Prevalence and risk factors for meticillin-resistant Staphylococcus aureus in an acute care hospital and long-term care facilities located in the same geographic area**. *Rev Esp Quimioter* 2014, **27**(3):190-195.

63. Budimir A, Pal MP, Bošnjak Z, Mareković I, Vuković D, Križan IR, Milas J, Plečko V, Kalenić S: **Prevalence and molecular characteristics of methicillin-resistant Staphylococcus aureus strains isolated in a multicenter study of nursing home residents in Croatia**. *Am J Infect Control* 2014, **42**(11):1197-1202.

64. March A, Aschbacher R, Pagani E, Sleghel F, Soelva G, Hopkins KL, Doumith M, Innocenti P, Burth J, Piazzani F *et al*: **Changes in colonization of residents and staff of a long-term care facility and an adjacent acute-care hospital geriatric unit by multidrug-resistant bacteria over a four-year period**. *Scand J Infect Dis* 2014, **46**(2):114-122.

65. Romaniszyn D, Pobiega M, Wójkowska-Mach J, Chmielarczyk A, Gryglewska B, Adamski P, Heczko PB, Ochońska D, Bulanda M: **The general status of patients and limited physical activity as risk factors of Methicillin-resistant Staphylococcus aureus occurrence in long-term care facilities residents in Krakow, Poland**. *BMC Infect Dis* 2014, **14**:271.

66. Ruscher C, Pfeifer Y, Layer F, Schaumann R, Levin K, Mielke M: **Inguinal skin colonization with multidrug-resistant bacteria among residents of elderly care facilities: Frequency, persistence, molecular analysis and clinical impact**. *Int J Med* 2014, **304**(8):1123-1134.

67. Stark L, Olofsson M, Löfgren S, Mölstad S, Lindgren PE, Matussek A: **Prevalence and molecular epidemiology of Staphylococcus aureus in Swedish nursing homes-As revealed in the SHADES study**. *Epidemiol Infect* 2014, **142**(6):1310-1316.

68. Gruber I, Heudorf U, Werner G, Pfeifer Y, Imirzalioglu C, Ackermann H, Brandt C, Besier S, Wichelhaus TA: **Multidrug-resistant bacteria in geriatric clinics, nursing homes, and ambulant care - Prevalence and risk factors**. *Int J Med* 2013, **303**(8):405-409.

69. Horner C, Parnell P, Hall D, Kearns A, Heritage J, Wilcox M: **Meticillin-resistant Staphylococcus aureus in elderly residents of care homes: Colonization rates and molecular epidemiology**. *J Hosp Infect* 2013, **83**(3):212-218.

70. Jans B, Schoevaerdts D, Huang TD, Berhin C, Latour K, Bogaerts P, Nonhoff C, Denis O, Catry B, Glupczynski Y: **Epidemiology of Multidrug-Resistant Microorganisms among Nursing Home Residents in Belgium**. *PLoS ONE* 2013, **8**(5).

71. Mossong J, Gelhausen E, Decruyenaere F, Devaux A, Perrin M, Even J, Heisbourg E: **Prevalence, risk factors and molecular epidemiology of methicillin- resistant Staphylococcus aureus (MRSA) colonization in residents of long-term care facilities in Luxembourg, 2010**. *Epidemiol Infect* 2013, **141**(6):1199-1206.

72. Van Der Donk CFM, Schols JMGA, Schneiders V, Grimm KH, Stobberingh EE: **Antibiotic resistance, population structure and spread of Staphylococcus aureus in nursing homes in the Euregion Meuse-Rhine**. *Eur J Clin Microbiol Infect Dis* 2013, **32**(11):1483-1489.

73. Andersson H, Lindholm C, Iversen A, Giske CG, Örtqvist Å, Kalin M, Fossum B: **Prevalence of antibiotic-resistant bacteria in residents of nursing homes in a Swedish municipality: Healthcare staff knowledge of and adherence to principles of basic infection prevention**. *Scand J Infect Dis* 2012, **44**(9):641-649.

74. Schoevaerdts D, Verroken A, Huang TD, Frennet M, Berhin C, Jamart J, Bogaerts P, Swine C, Glupczynski Y: **Multidrug-resistant bacteria colonization amongst patients newly admitted to a geriatric unit: a prospective cohort study**. *J Infect* 2012, **65**(2):109-118.

75. Olofsson M, Lindgren PE, Östgren CJ, Midlöv P, Mölstad S: **Colonization with Staphylococcus aureus in Swedish nursing homes: A cross-sectional study**. *Scand J Infect Dis* 2012, **44**(1):3-8.

76. Pfingsten-Würzburg S, Pieper DH, Bautsch W, Probst-Kepper M: **Prevalence and molecular epidemiology of meticillin-resistant Staphylococcus aureus in nursing home residents in northern Germany**. *J Hosp Infect* 2011, **78**(2):108-112.

77. Schwaber MJ, Masarwa S, Navon-Venezia S, Kandlik Y, Chmelnitsky I, Smollan G, Glick R, Neria G, Carmeli Y: **High prevalence of methicillin-resistant Staphylococcus aureus among residents and staff of long-term care facilities, involving joint and parallel evolution**. *Clin Infect Dis* 2011, **53**(9):910-913.

78. Lasseter G, Charlett A, Lewis D, Donald I, Howell-Jones R, McNulty CA: **Staphylococcus aureus carriage in care homes: identification of risk factors, including the role of dementia**. *Epidemiol Infect* 2010, **138**(5):686-696.

79. March A, Aschbacher R, Dhanji H, Livermore DM, Böttcher A, Sleghel F, Maggi S, Noale M, Larcher C, Woodford N: **Colonization of residents and staff of a long-term-care facility and adjacent acute-care hospital geriatric unit by multiresistant bacteria**. *Clin Microbiol Infect* 2010, **16**(7):934-944.

80. Baldwin NS, Gilpin DF, Hughes CM, Kearney MP, Gardiner DA, Cardwell C, Tunney MM: **Prevalence of methicillin-resistant staphylococcus aureus colonization in residents and staff in nursing homes in Northern Ireland**. *J Am Geriatr Soc* 2009, **57**(4):620-626.

81. Denis O, Jans B, Deplano A, Nonhoff C, De Ryck R, Suetens C, Struelens MJ: **Epidemiology of methicillin-resistant Staphylococcus aureus (MRSA) among residents of nursing homes in Belgium**. *J Antimicrob Chemother* 2009, **64**(6):1299-1306.

82. Brugnaro P, Fedeli U, Pellizzer G, Buonfrate D, Rassu M, Boldrin C, Parisi SG, Grossato A, Palù G, Spolaore P: **Clustering and risk factors of methicillin-resistant staphylococcus aureus carriage in two italian long-term care facilities**. *Infection* 2009, **37**(3):216-221.

83. Eveillard M, Charru P, Rufat P, Hippeaux MC, Lancien E, Benselama F, Branger C: **Methicillin-resistant Staphylococcus aureus carriage in a long-term care facility: Hypothesis about selection and transmission**. *Age Ageing* 2008, **37**(3):294-299.

84. Manzur A, Gavalda L, Ruiz de Gopegui E, Mariscal D, Dominguez MA, Perez JL, Segura F, Pujol M: **Prevalence of methicillin-resistant Staphylococcus aureus and factors associated with colonization among residents in community long-term-care facilities in Spain**. *Clin Microbiol Infect* 2008, **14**(9):867-872.

85. Pop-Vicas A, Mitchell SL, Kandel R, Schreiber R, D'Agata EMC: **Multidrug-resistant gram-negative bacteria in a long-term care facility: Prevalence and risk factors**. *J Am Geriatr Soc* 2008, **56**(7):1276-1280.

86. Smith CS, Parnell P, Hodgson G, Darby B, Barr B, Tompkins D, Heritage J, Wilcox MH: **Are methicillin-resistant Staphylococcus aureus that produce Panton-Valentine leucocidin (PVL) found among residents of care homes?** *J Antimicrob Chemother* 2008, **62**(5):968-972.

87. Barr B, Wilcox MH, Brady A, Parnell P, Darby B, Tompkins D: **Prevalence of methicillin-resistant Staphylococcus aureus colonization among older residents of care homes in the United Kingdom**. *Infect Control Hosp Epidemiol* 2007, **28**(7):853-859.

88. Kerttula AM, Lyytikäinen O, Virolainen A, Finne-Soveri H, Agthe N, Vuopio-Varkila J: **Staphylococcus aureus colonization among nursing home residents in a large Finnish nursing home**. *Scand J Infect Dis* 2007, **39**(11-12):996-1001.

89. Daeschlein G, Assadian O, Rangous I, Kramer A: **Risk factors for Staphylococcus aureus nasal carriage in residents of three nursing homes in Germany**. *J Hosp Infect* 2006, **63**(2):216-220.

90. Suetens C, Niclaes L, Jans B, Verhaegen J, Schuermans A, Van Eldere J, Buntinx F: **Methicillin-resistant Staphylococcus aureus colonization is associated with higher mortality in nursing home residents with impaired cognitive status**. *J Am Geriatr Soc* 2006, **54**(12):1854-1860.

91. Cretnik TZ, Vovko P, Retelj M, Jutersek B, Harlander T, Kolman J, Gubina M: **Prevalence and nosocomial spread of methicillin-resistant Staphylococcus aureus in a long-term-care facility in Slovenia**. *Infect Control Hosp Epidemiol* 2005, **26**(2):184-190.

92. Vovko P, Retelj M, Cretnik TZ, Jutersek B, Harlander T, Kolman J, Gubina M: **Risk factors for colonization with methicillin-resistant Staphylococcus aureus in a long-term-care facility in Slovenia**. *Infect Control Hosp Epidemiol* 2005, **26**(2):191-195.

93. Mendelson G, Yearmack Y, Granot E, Ben-Israel J, Colodner R, Raz R: **Staphylococcus aureus carrier state among elderly residents of a long-term care facility**. *J Am Med Dir Assoc* 2003, **4**(3):125-127.

94. von Baum H, Schmidt C, Svoboda D, Bock-Hensley O, Wendt C: **Risk factors for methicillin-resistant Staphylococcus aureus carriage in residents of German nursing homes**. *Infect Control Hosp Epidemiol* 2002, **23**(9):511-515.

95. Hoefnagels-Schuermans A, Niclaes L, Buntinx F, Suetens C, Jans B, Verhaegen J, Van Eldere J: **Molecular epidemiology of methicillin-resistant Staphylococcus aureus in nursing homes: A cross-sectional study**. *Infect Control Hosp Epidemiol* 2002, **23**(9):546-549.

96. O'Sullivan NR, Keane CT: **The prevalence of methicillin-resistant staphylococcus aureus among the residents of six nursing homes for the elderly**. *J Hosp Infect* 2000, **45**(4):322-329.

97. Cox RA, Bowie PES: **Methicillin-resistant Staphylococcus aureus colonization in nursing home residents: A prevalence study in Northamptonshire**. *J Hosp Infect* 1999, **43**(2):115-122.

98. Niclaes L, Buntinx F, Banuro F, Lesaffre E, Heyrman J: **Consequences of MRSA carriage in nursing home residents**. *Epidemiol Infect* 1999, **122**(2):235-239.

99. Rahimi AR: **Prevalence and outcome of methicillin-resistant staphylococcus aureus colonization in two nursing centers in Georgia**. *J Am Geriatr Soc* 1998, **46**(12):1555-1557.

100. Fraise AP, Mitchell K, O'Brien SJ, Oldfield K, Wise R: **Methicillin-resistant Staphylococcus aureus (MRSA) in nursing homes in a major UK city: An anonymized point prevalence survey**. *Epidemiol Infect* 1997, **118**(1):1-5.

101. Wong S-C, Chen J-K, Yuen L-H, Chan V-M, AuYeung C-Y, Leung S-M, So S-C, Chan B-K, Li X, Leung J-Y: **Air dispersal of meticillin-resistant Staphylococcus aureus in residential care homes for the elderly: implications for transmission during the COVID-19 pandemic**. *Journal of Hospital Infection* 2022, **123**:52-60.

102. Huang YC, Chen CJ, Lauderdale TLY: **Detection, spread and phylogeny of meticillin-resistant Staphylococcus aureus sequence type 45 in Taiwan**. *Microb Genom* 2021, **7**(4):NA.

103. He W-P, Gu F-F, Zhang J, Li X-X, Xiao S-Z, Zeng Q, Ni Y-X, Han L-Z: **Molecular characteristics and risk factor analysis of Staphylococcus aureus colonization put insight into CC1 colonization in three nursing homes in Shanghai**. *Plos one* 2021, **16**(10):e0253858.

104. Chow A, Htun HL, Hon PY, Ang B, Kanagasabai K, Koh J, Holden MTG, Hsu LY: **Comparative epidemiology and factors associated with major healthcare-associated methicillin-resistant Staphylococcus aureus clones among interconnected acute-, intermediate- and long-term healthcare facilities in Singapore**. *Clin Microbiol Infect* 2020.

105. Sasahara T, Ae R, Yoshimura A, Kosami K, Sasaki K, Kimura Y, Akine D, Ogawa M, Hamabata K, Hatakeyama S *et al*: **Association between length of residence and prevalence of MRSA colonization among residents in geriatric long-term care facilities**. *BMC Geriatr* 2020, **20**(1):481.

106. Htun HL, Hon PY, Holden MTG, Ang B, Chow A: **Chlorhexidine and octenidine use, carriage of qac genes, and reduced antiseptic susceptibility in methicillin-resistant Staphylococcus aureus isolates from a healthcare network**. *Clin Microbiol Infect* 2019, **25**(9):1154.e1151-1154.e1157.

107. Chen H, Au KM, Hsu KE, Lai CKC, Myint J, Mak YF, Lee SY, Wong TY, Tsang NC: **Multidrug-resistant organism carriage among residents from residential care homes for the elderly in Hong Kong: A prevalence survey with stratified cluster sampling**. *Hong Kong Med J* 2018, **24**(4):350-360.

108. Lee CM, Lai CC, Chiang HT, Lu MC, Wang LF, Tsai TL, Kang MY, Jan YN, Lo YT, Ko WC *et al*: **Presence of multidrug-resistant organisms in the residents and environments of long-term care facilities in Taiwan**. *J Microbiol Immunol Infect* 2017, **50**(2):133-144.

109. Gu FF, Zhang J, Zhao SY, Yang ZR, Zhang YL, Xiao SZ, Wang S, Guo XK, Qu JM, Ni YX *et al*: **Risk factors for methicillin-resistant Staphylococcus aureus carriage among residents in 7 nursing homes in Shanghai, China**. *Am J Infect Control* 2016, **44**(7):805-808.

110. Tsao F-Y, Kou H-W, Huang Y-C: **Dissemination of methicillin-resistant Staphylococcus aureus sequence type 45 among nursing home residents and staff in Taiwan**. *Clin Microbiol Infect* 2015, **21**(5):451-458.

111. Zhang J, Gu FF, Zhao SY, Xiao SZ, Wang YC, Guo XK, Ni YX, Han LZ: **Prevalence and molecular epidemiology of staphylococcus aureus among residents of seven nursing homes in Shanghai**. *PLoS ONE* 2015, **10**(9).

112. Chuang VW, Tsang IH, Keung JP, Leung JY, Yuk JM, Wong DK, Au SS, Tam RK, Lam WW, Kwan MC *et al*: **Infection control intervention on meticillin resistant Staphylococcus aureus transmission in residential care homes for the elderly**. *J Infect Prev* 2015, **16**(2):58-66.

113. Lim CJ, Cheng AC, Kennon J, Spelman D, Hale D, Melican G, Sidjabat HE, Paterson DL, Kong DC, Peleg AY: **Prevalence of multidrug-resistant organisms and risk factors for carriage in long-term care facilities: a nested case-control study**. *J Antimicrob Chemother* 2014, **69**(7):1972-1980.

114. Cheng VC, Tai JW, Wong ZS, Chen JH, Pan K, Hai Y, Ng W-C, Chow DM, Yau MC, Chan JF: **Transmission of methicillin-resistant Staphylococcus aureus in the long term care facilities in Hong Kong**. *BMC Infect Dis* 2013, **13**(1):1-10.

115. Ho PL, Lai EL, Chow KH, Chow LSM, Yuen KY, Yung RWH: **Molecular epidemiology of methicillin-resistant Staphylococcus aureus in residential care homes for the elderly in Hong Kong**. *Diagn Microbiol Infect Dis* 2008, **61**(2):135-142.

116. Ho PL, Wang TKF, Ching P, Mak GC, Lai E, Yam WC, Seto WH: **Epidemiology and genetic diversity of methicillin-resistant Staphylococcus aureus strains in residential care homes for elderly persons in Hong Kong**. *Infect Control Hosp Epidemiol* 2007, **28**(6):671-678.

117. ISHIHARA K, ADACHI M, EGUCHI J, WASHIZU M, KOSUGI M, OKUDA K: **Prevalence of Staphylococcus species and Candida albicans in the oral cavities of elderly who require daily care in a nursing home**. *Bull Tokyo Dent* 2000, **41**(4):169-174.

118. Washio M, Nishisaka S, Kishikawa K, Irie K, Shinkawa A, Tashiro T, Nohtomi A, Hamada T, Okayama M, Makita Y *et al*: **Incidence of methicillin-resistant Staphylococcus aureus (MRSA) isolation in a skilled nursing home: a third report on the risk factors for the occurrence of MRSA infection in the elderly**. *J Epidemiol* 1996, **6**(2):69-73.
